# Measuring social desirability bias in a multi-ethnic cohort sample: its relationship with self-reported physical activity, dietary habits, and factor structure

**DOI:** 10.1186/s12889-023-15309-3

**Published:** 2023-03-01

**Authors:** Wen Lin Teh, Edimansyah Abdin, Asharani P.V., Fiona Devi Siva Kumar, Kumarasan Roystonn, Peizhi Wang, Saleha Shafie, Sherilyn Chang, Anitha Jeyagurunathan, Janhavi Ajit Vaingankar, Chee Fang Sum, Eng Sing Lee, Rob M. van Dam, Mythily Subramaniam

**Affiliations:** 1 Research Division, Institute of Mental Health, 10 Buangkok View, Singapore, Singapore; 2 Admiralty Medical Centre, Khoo Teck Puat Hospital, 676 Woodlands Drive 71, Singapore, Singapore; 3 National Healthcare Group Polyclinics, 3 Fusionopolis Link. Nexus@One-North, Singapore, Singapore; 4 Lee Kong Chian School of Medicine, Nanyang Technological University, Singapore, Singapore; 5 Saw Swee Hock School of Public Health, National University of Singapore, 12 Science Drive 2, Singapore, Singapore; 6 Departments of Exercise and Nutrition Sciences and Epidemiology, Milken Institute School of Public Health, The George Washington University, 950 New Hampshire Ave NW, Washington, DC United States

**Keywords:** Marlowe-crowne, MCSDS, Social desirability, Multi-ethnic, Asia, CFA

## Abstract

**Background:**

Social desirability bias is one of the oldest forms of response bias studied in social sciences. While individuals may feel the need to fake good or bad answers in response to sensitive or intrusive questions, it remains unclear how rampant such a bias is in epidemiological research pertaining to self-reported lifestyle indicators in a multicultural Asian context. The main purpose of the current study is, therefore, to examine the sociodemographic correlates and impact of social desirability responding on self-reported physical activity and dietary habits at an epidemiological scale in a non-western multi-cultural Asian setting.

**Methods:**

Prior to the main analyses, confirmatory and exploratory factor analyses were conducted to determine the factorial validity of a western derived concept of social desirability. Multiple regression analyses were conducted on cross-sectional data (n = 2995) extracted from a nationwide survey conducted between 2019 and 2020.

**Results:**

A unique factor structure of social desirability was found and was therefore used for subsequent analyses. Multiple regression analyses revealed older age groups, the Indian ethnic group, those with past or present marriages, and having no income, had a significantly greater tendency to act on the bias.

**Conclusion:**

The construct of social desirability bias was fundamentally different in a multicultural context than previously understood. Only a small proportion of variance of self-report lifestyle scores was explained by social desirability, thus providing support for data integrity.

## Introduction

One of the oldest and most common forms of response bias studied in social science, the social desirability bias, is defined as an individual’s propensity to respond in a way that is viewed favourably by society. Alike many forms of response biases, social desirability bias causes misreports, either by faking good or bad responses, making the scientific inquiry of any social phenomenon challenging [1].

Self-report surveys, which are commonly used in attitudinal research, are particularly vulnerable to such bias. Surveys that ask of sensitive or intrusive topics increases an individual’s tendency to misreport for fear of social disapproval or embarrassment. The tendency to misreport depends greatly on the sensitivity of the context, and the magnitude of its confounding effect therefore varies. While there are alternatives to self-report measures that can improve the precision of data, such as dietary or physical activity surveillance used in health research, these alternatives incur larger administrative costs, participant burden, and intrusion [2, 3]. Self-report thus remains a popular method due to its ease of administration, minimal costs, and greater nonmaleficence.

What is considered to be socially acceptable depends largely on culture. Since such a bias is greatly attributed by sociocultural norms, past research that are typically based on homogeneous and individualistic cultures may not be generalizable to a multi-cultural society in Asia. Collectivistic societies that strive for cooperativeness and sensitivity may exercise more of such a bias than individualistic societies [4]. However, empirical data on the effects of social desirability bias is lacking in collectivistic societies. Similarly, it is also unclear if western-derived constructs of social desirability can be generalized to a non-western population that is also multi-cultural [5].

Social desirability bias has been largely neglected in aspects of attitudinal research where there is a greater cause for concern of misreporting. Misreporting on lifestyle issues, such as smoking, physical exercise, or dietary habits, may in turn confound precision greatly needed to inform health policy [6–8]. Research has shown that social desirability bias is readily exercised by children and adults alike on self-reporting dietary intake for instance [9, 10]. Individuals with a less than ideal height or weight tend to self-report within the range deemed to be socially acceptable [11]. Similarly, there is also a tendency to over-report physical activity levels to a significant degree [12].

In Singapore, epidemiological research on physical and mental health are becoming increasingly apparent. To name a few: the national population health survey (NPSH) conducted by the Ministry of Health that began in 1998 [13], the national youth survey (NYS) commenced in 2002 [14], the Well-being of the Singapore Elderly (WiSE) population study which involved older persons above the age of 60 started its first iteration in 2010 [15], the Singapore Mental Health Study (SMHS) which focused on nation-wide mental health surveillance, was initiated in 2009 [16], and finally, the Knowledge, Attitudes and Practices (KAP) survey on diabetes commenced in 2019 [17]; all of which are ongoing epidemiological research with a central aim of providing up-to-date representative data on the health and well-being of Singapore’s population. It is crucial to note that none of the existing local health research have studied the impact of social desirability bias. At this juncture, not enough is understood of the effects of such a bias on self-reported physical health and lifestyle concerns—indicators that are typically captured in existing local epidemiological research. If such effects exist, how much of self-report data is attributed to such a bias and which groups have a greater tendency to exercise such a bias? Understanding the direction and magnitude of the bias is thus necessary so as to address it.

Singapore is a multi-ethnic and multi-cultural city-state in Southeast Asia with a population of approximately 4 million residents (citizens and permanent residents; based on 2021 data from the Singapore Department of Statistics). To the best of knowledge, this is the first study to report the sociodemographic correlates of social desirability bias and its impact on self-reported lifestyle behaviours—dietary habits and physical activity specifically. Results of the study will be beneficial for understanding the current level of bias, if any, in a multi-ethnic Asian population.

## Methods

Data were drawn from a large cross-sectional study called the Knowledge, Attitudes, and Practices (KAP) study, which is a nationwide survey conducted between 2019 and 2020 to understand the KAP of diabetes in relation to lifestyle factors in Singapore. The full details of the study’s protocol and design have been published elsewhere [17]. Briefly, Singapore residents were randomly selected from a national administrative registry and were subsequently approached by trained interviewers at their households to complete a set of questionnaires. An additional hundred diabetes cases were conveniently sampled. All participants completed written informed consent and were compensated an amount for their participation. An overall response rate of 66.2% was achieved, which was expected result [17, 18]. Chi-square analyses between responders and non-responders were conducted to ascertain response bias. Based on the original sample size of 2895, younger age groups (χ^2^(3) = 20.70, *p* < .001), those of Malay and Indian ethnicities (χ^2^(3) = 57.5110, *p* < .001), were more likely to respond to the survey. The study was approved by the relevant local ethics committees: Institutional Research Review Committee (IRRC) and the National Healthcare Group Domain Specific Review Board (DSRB; Ref no. 2018/00430).

### Participants

The final study sample included individuals from all ethnic groups (Chinese, Malay, Indian, or other), who were 18 years of age and above, and were literate in any of Singapore’s national languages: English, Chinese, Malay, or Tamil. The study excluded individuals who were not living in Singapore, hospitalised or institutionalised during the study period. Data from a total of 2995 individuals (including 100 cases with diabetes) were extracted for the purpose of this investigation.

### Measures

Marlowe–Crowne Social Desirability Scale (MCSDS) by Crowne and Marlowe (1960) measures the level of social desirability bias [19]. Participants were asked to make True/False responses to 33 items. Individual item responses range from 1 to 2. However, item 1, “*Before voting I thoroughly investigate the qualifications of all the candidates*,” was dropped from the questionnaire after participants expressed concerns that it assessed their political preferences and may be used for other purposes. ‘False’ responses were denoted as a 2, and ‘True’ responses were given the value 1. The overall internal consistency of 32 items was found to be good (*α* = 0.75). The short-form versions (type A, B, C, XX, X1, X2) were extracted from the original 33-item. Items 1, 2, 4, 7, 8, 13, 16, 17, 18, 20, 21, 24, 25, 26, 27, 29, 31, and 33 were reversed scored. Higher scores denote a greater bias.

Global Physical Activity Questionnaire (GPAQ), developed by the World Health Organisation (WHO, 2006), measures self-reported levels of physical activity (moderate or vigorous intensity levels) on average at work, travelling to and from places, during recreational activities on average daily. It also records self-reported minutes spent on sedentary activities per day, such as sitting or reclining while awake [20]. Metabolic Equivalents (METs) are calculated by the ratio of working to resting metabolic rate in minutes/week for each domain. An overall global MET score (GPAQ grand MET score) was subsequently calculated by the sum of METs in each domain except the sedentary domain, which was calculated separately (GPAQ sedentary score).

A locally validated diet screener was utilised to record the level of consumption of typical food and beverages in the past one year [21]. Respondents self-reported the frequency of food/beverage intake on a 10-point scale, ranging from “never/rarely” to “6 or more times per day”. A Dietary Approaches to Stop Hypertension (DASH) score—a sensitive measure to test against health-related outcomes [21]—was subsequently calculated by the amount of intake of vegetables, nuts or legumes, fruit, low fat dairy, processed or red meat (reversed scored), whole grains, and sweetened beverages (reversed scored) as a whole (based on standard servicing sizes). Participants eventually received a score of 1 to 5 on each of the seven categories depending on the quintile level of intake [22, 23], which were eventually totalled as an overall DASH score. Higher DASH scores thus represent a healthier diet.

A sociodemographic questionnaire comprising of age, gender, ethnicity, personal monthly income (in Singapore dollars (SGD), highest education level, height and weight information were also completed by the participants.

### Statistical analysis

As the MCSDS scale was originally conceptualized in the west, it is unclear if the original factorial structure is valid in a multicultural Asian setting, as factors of social desirability depend heavily on cultural norms that determine what constitutes a desirable response. Past research showed that the MCSDS scale, while internally valid and reliable, do not have a consistent factorial structure across countries. While the original one-factor solution has been supported in the Icelandic population [24], various two-factor solutions have gained greater support, such as in Africa, Switzerland, Canada, and in Malaysia [25–27]. Additionally, abbreviated versions of the scale (e.g., type A, B, C, XX, X1, X2) that contain 10 to 20 of the original 33 items [28, 29], and that were created out of a need to address the lack of factorial fit and lengthiness of the scale, have also been widely validated [30, 31]. Generally, the assumed single-dimensionality of alternative forms too have received mixed support [24, 32]. Thus, prior to the main analyses, confirmatory and exploratory factor analyses were conducted to ascertain the factorial fit of MCSDS in the local sample.

### Confirmatory factor analysis (CFA)

Missing data accounted for 2.33% (7/2995) of all data and were deleted listwise. All analyses were conducted based on the final sample size of *n* = 2988. To address the dichotomous True/False format of the MCSDS, CFA was performed using unweighted least squares estimator (ULSMV), set at a default mode of 1000 iterations and delta parameterization. A fixed factor method for factor scaling was used by fixing the model factor variance to 1 and constraints were freed on the first item (on item 2, because item 1 was dropped) of each factor [33]. Model fit was assessed using absolute and relative fit indices, i.e., model chi-square χ^2^, root mean square error of approximation (RMSEA), comparative fit index (CFI), and Tucker–Lewis index (TLI). This study utilised Marsh et al.’s recommendations for good fit as the model evaluation criteria [34]: RMSEA < 0.08; CFI > 0.90; TLI > 0.90.

### Exploratory factor analysis (EFA)

EFA was subsequently performed on one split-half of the sample (*n* = 1494) to extract the factor structure of MCSDS that gave the best fit of data. Similarly, EFA was performed using unweighted least square mean and variance (ULSMV), with a default setting of oblique geomin rotation. The final model derived from EFA was tested using CFA on the second split-half sample, and eventually, on the overall sample. Communality coefficients (h^2^) were calculated by the formula: 1 - *estimated residual variances*. CFA and EFA were performed on Mplus 8.3. by Muthén and Muthén [35].

### Multiple linear regression analyses

To address the main aim of the study, multiple linear regression analysis was performed on 2895 data points (with 100 cases with diabetes removed) between sociodemographic categorical variables, such as age, gender, ethnicity, personal income, and education levels, and the total continuous scores of the 21-item adapted version of the MCSDS. Subsequent hierarchical linear regression analyses using the enter method were performed to ascertain the unique contribution of MCSDS in predicting dietary habits, physical activity, and sedentary behaviours. Sociodemographic and BMI covariates were entered in the first step to control for confounds, followed by the modified MCSDS which was added in the second step. Weights were applied to the survey data for all multiple linear regression analysis for representativeness. All missing values were deleted listwise. The analyses were conducted on IBM SPSS statistics software version 23.0.

## Results

### Goodness of fit of original, popular short forms, and the modified version

**Fig. 1:**
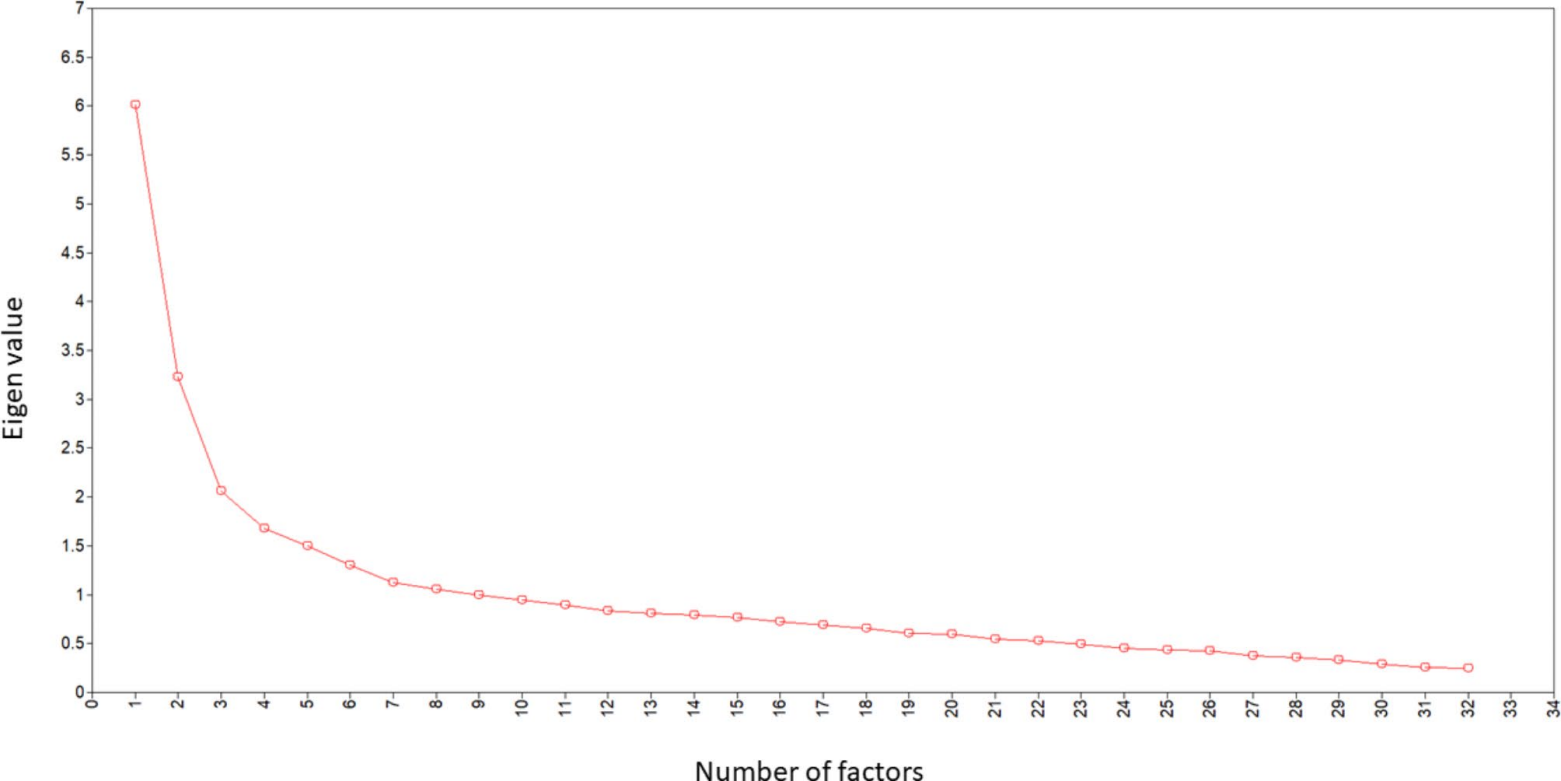
Extracted eigen values from Exploratory Factor Analysis of the Marlowe-Crowne Social Desirability Scale (MCSDS; the first item of the 33 items was removed due to participant feedback)

After dropping the first item of the scale due to participants’ concerns, CFA was performed on the remaining 32 items, and type A, B, C, XX, X1, X2 short-form versions, to determine the factorial appropriateness of existing popular forms. Model parameters of all full and abbreviated forms of the MCSDS derived from the CFA were unsatisfactory (Table 1). This led the team to conduct EFA on the full 32-item scale (with item 1 dropped) using a split-half sample. The number of factors extracted was determined by an inspection of the scree plot [36], which suggested an optimal extraction of three factors (Fig. 1). Items that had weak rotated factor coefficients (< 0.04) were removed (see Table 2). This was proceeded by additional EFA iterations to eliminate problematic items (i.e., low factor loadings and cross-loadings). Afterwards, it was realised that the third factor could not be identified due to insufficient component saturation and common meaning between its two items, leading author WLT to reconsider a two-factor solution. The three-factor solution was thus rejected and multiple EFAs were subsequently performed to eliminate problematic items in the two-factor solution. The degree of stability of the final two-factor model was confirmed by performing CFA on the second split-half of the sample [37]. Factor coefficients with greater than 0.40 item loadings were retained, resulting in the final retention of 21 items (Table 2). The final 21-item two-factor solution was assessed with CFA on the second split-half and eventually on the full sample (Table 1). Parameter estimates of the final 21-item two-factor solution showed vast improvements to the model fit and represented the best fit of data.

**Table 1:** Exploratory (EFA) and Confirmatory Factor Analysis (CFA) derived Absolute and Comparative fit measures of original and short form versions of the Marlowe-Crowne Social Desirability Scale (MCSDS)

						RMSEA			
s/n		Factor	*n*	χ2	SRMR	estimate	90% CI	CFI	TLI
1	32-item	One factor	2988	3777.5*	0.960	0.0490	0.047, 0.050	0.682	0.660
2	32-item (Paulhus and Reid, 1989)	Two factors	2988	4920.3*	0.111	0.0570	0.055, 0.058	0.572	0.545
3	11-item (short-form A; Reynolds, 1982)	One factor	2988	421.8*	0.073	0.0540	0.049, 0.058	0.849	0.812
4	12-item (short-form B; Reynolds, 1982)	One factor	2988	523.9*	0.075	0.0540	0.050, 0.058	0.844	0.810
5	13-item (short-form C; Reynolds, 1982)	One factor	2988	703.4*	0.081	0.0570	0.054, 0.061	0.816	0.779
6	20-item (short-form XX; Strahan and Gerbasi, 1972)	One factor	2988	1387.3*	0.090	0.0490	0.047, 0.051	0.770	0.743
7	10-item (short-form X1; Strahan and Gerbasi, 1972)	One factor	2988	280.9*	0.074	0.0480	0.043, 0.054	0.826	0.777
8	10-item (short-form X2; Strahan and Gerbasi, 1972)	One factor	2988	267.2*	0.073	0.0470	0.042, 0.052	0.834	0.786
9	14-item^a^ (Ramanaiah et al., 1977)	Two factors	2988	886.3*	0.095	0.0600	0.056, 0.063	0.678	0.614
10	21-item	Two factors (CLI, & IIM)	1494^a^	481.7*	0.068	0.032	0.029, 0.036	0.915	0.905
11	21-item	Two factors (CLI, & IIM)	2988	812.5*	0.061	0.033	0.031, 0.036	0.917	0.907
12	21-item	Two factors (CLI, & IIM)	1494^a^	418.9*	0.060	0.031	0.028, 0.035	0.931	0.914

note: s/n 1–11 denote parameter estimates from CFA; s/n 12 denote parameter estimates from EFA; item 1 was removed from all analyses due to participants’ concerns; 14-item^a^: Item 1 was excluded, thus CFA was conducted with the remaining 14 items of the 15-item version by Ramanaiah et al.; CLI: Civil & Lawful Impression; IIB Integrous Image Boosting; the modified 21-item version (s/n 10–12) was based on the final EFA two-factor solution that best fit of current data; 1494^a^ represents the split-half sample size; χ2 refers to the model chi-square result; RMSEA is the root mean square error of approximation; CFI refers to the comparative fit index; TLI refers to Tucker–Lewis index; SRMR is the standardized root mean squared residual index; 90% CI refers to the 90% confidence interval; values indicated with the asterisk * sign represents significant chi-square results;

**Table 2:** Final 21-item modified Marlowe-Crowne Social Desirability Scale (MCSDS) Factor Structure Matrix Rotated to the Geomin Criterion

		Factor 1	Factor 2	h^2^
Item in the full MCSDS	Item description:	Civil & Lawful impression	Integrous Image boosting	
3	It is sometimes hard for me to go on with my work if I am not encouraged	**0.520**	-0.174	0.244
4	I have never intensely disliked anyone. (T)	**0.601**	-0.113	0.331
6	I sometimes feel resentful when I don’t get my way.	**0.677**	-0.151	0.417
9	If I could get into a movie without paying and be sure I was not seen, I would probably do it.	**0.511**	0.028	0.27
10	On a few occasions, I have given up something because I thought too little of my ability.	**0.517**	-0.096	0.246
12	There have been times when I felt like rebelling against people in authority even though I knew they were right.	**0.599**	-0.003	0.358
14	I can remember “playing sick” to get out of something.	**0.560**	0.175	0.405
15	There have been occasions when I have taken advantage of someone	**0.499**	0.257	0.395
19	I sometimes try to get even rather than forgive and forget.	**0.453**	0.089	0.238
22	At times I have really insisted on having things my own way.	**0.442**	-0.061	0.183
23	There have been occasions when I felt like smashing things.	**0.545**	0.116	0.35
28	There have been times when I was quite jealous of the good fortune of others.	**0.649**	0.081	0.46
30	I am sometimes irritated by people who ask favors of me.	**0.442**	0.111	0.239
5	On occasions I have had doubts about my ability to succeed in life.	0.06	**0.496**	0.268
8	My table manners at home are as good as when I eat out in a restaurant. (T)	0.153	**0.433**	0.252
13	No matter who I’m talking to, I’m always a good listener. (T)	-0.009	**0.688**	0.47
16	I’m always willing to admit it when I make a mistake. (T)	0.115	**0.510**	0.309
17	I always try to practice what I preach. (T)	0.004	**0.523**	0.275
21	I am always courteous, even to people who are disagreeable. (T)	-0.008	**0.632**	0.396
29	I have almost never felt the urge to tell someone off. (T)	-0.051	**0.427**	0.171
33	I have never deliberately said something that hurt someone’s feelings. (T)	0.135	**0.486**	0.296

note: item 1 was dropped due to concerns made by participants during data collection; coefficients more than |0.40| are in bold and retained; items 2,7,11,18,20,24,25,26,27,31,32 of the original MCSDS were removed due to low coefficients less than |0.40| or cross-loadings; all items were significant; h^2^ is the communality coefficient; (T) represents “True” responses, and is thus reverse scored.

### The modified 21-item version (modified MCSDS)

The final adapted MCSDS comprised two factors with 21 items (Table 2). The first factor—Civil and Lawful Impression—centralises on giving an impression of being seen as non-hostile (to an authority) or law-abiding. The second factor—Integrous Image boosting—centralises on presenting oneself with an honest, upright, or refined image. The 21-item MCSDS is hereafter referred to as the modified MCSDS. The internal consistency of the modified MCSDS was found to be acceptable, *α* = 0.74. The correlation between the full MCSDS and the modified version was *r* = .938, with *r*^*2*^ *=* 0.890.

### Sociodemographic correlates of the modified MCSDS

The overall model was significant, *F*(19, 2729) = 14.36, *p* < .001, R^2^ = 0.168. Multiple linear regression analyses revealed that older age (35 years and above) as compared to younger age (below 35 years), Indian ethnicity as compared to the Chinese ethnicity, marriage history (current and past) as compared to single/never married, and having no income as compared to below 2k and 10k and above income brackets, were significantly associated with higher social desirability bias. Gender, education levels, Malay, and other ethnicities, did not emerge as significant sociodemographic factors of the bias. The results of the multiple weighted linear aggression analyses are summarized in Table 3.

**Table 3:** Sociodemographic determinants of the modified Marlowe-Crowne Social Desirability Scale (MCSDS).

			Factor 1: Civil and Lawful Impression				Factor 2: Integrous Image Boosting				21-item MCSDS (Total)			
				95% CI				95% CI				95% CI		
Variables	n	%	*b*	lower	upper	*p*-value	*b*	lower	upper	*p*-value	*b*	lower	upper	*p*-value
Age (in years)														
35 to 49	719	28.2	0.75	0.24	1.28	**0.004**	0.4	0.14	0.66	**0.003**	1.16	0.49	1.82	**0.001**
50 to 64	774	26.8	1.7	1.16	2.24	**< 0.001**	0.91	0.62	1.2	**< 0.001**	2.61	1.92	3.29	**< 0.001**
65 and above	579	15.1	1.82	1.17	2.47	**< 0.001**	1.08	0.76	1.4	**< 0.001**	2.9	2.1	3.7	**< 0.001**
18 to 34 (ref)	823	29.9	0											
Gender														
Male	1421	47.5	-0.12	-0.42	0.18	0.44	0.103	-0.06	0.27	0.211	-0.02	-0.41	0.37	0.93
Female (ref)	1474	51.6	0											
Ethnicity														
Malay	974	12.7	-0.03	-0.33	0.27	0.85	0.37	0.23	0.52	**< 0.001**	0.35	-0.03	0.72	0.072
Indian	918	8.6	0.06	-0.22	0.34	0.66	0.36	0.22	0.51	**< 0.001**	0.43	0.07	0.78	**0.02**
Other	207	2.9	0.13	-0.41	0.67	0.63	0.3	0.02	0.57	**0.033**	0.43	-0.28	1.14	0.233
Chinese (ref)	796	75.8	0											
Highest Education														
Primary or lower	637	20.4	0.02	-0.54	0.58	0.94	0.16	-0.14	0.45	0.293	0.18	-0.52	0.88	0.62
Secondary	684	20.3	-0.45	-0.97	0.07	0.09	0.15	-0.12	0.41	0.281	-0.31	-0.95	0.34	0.35
Pre-U/Junior College	126	4.8	-0.1	-0.77	0.58	0.78	0.1	-0.27	0.47	0.58	0.01	-0.9	0.92	0.986
Vocational Institute/ITE	267	6.6	0.18	-0.5	0.86	0.6	0.44	0.09	0.78	**0.013**	0.62	-0.28	1.51	0.176
Diploma	479	18.5	-0.11	-0.6	0.38	0.66	0.33	0.08	0.58	**0.011**	0.22	-0.42	0.86	0.506
Tertiary and above (ref)	702	29.5	0											
Marital Status														
Currently married/cohabiting	1860	61.7	0.55	0.07	1.03	**0.025**	0.53	0.27	0.78	**< 0.001**	1.08	0.46	1.69	**0.001**
Previously married	303	9.1	0.94	0.28	1.59	**0.005**	0.69	0.36	1.02	**< 0.001**	1.63	0.82	2.43	**< 0.001**
Never married (ref)	731	29.2	0											
Personal Monthly Income														
Below 2k	1236	40.1	-0.19	-0.73	0.36	0.5	-0.49	-0.75	-0.23	**< 0.001**	-0.68	-1.34	-0.012	**0.046**
2k to 3999	698	25.1	0.11	-0.47	0.68	0.71	-0.49	-0.79	-0.19	**0.001**	-0.38	-1.1	0.34	0.298
4k to 5999	318	13.4	0.08	-0.62	0.78	0.82	-0.45	-0.8	-0.1	**0.011**	-0.37	-1.24	0.51	0.41
6k to 9999	183	8.2	-0.32	-1.07	0.44	0.41	-0.49	-0.88	-0.09	**0.016**	-0.8	-1.77	0.16	0.104
10k and above	117	5.9	-0.49	-1.39	0.4	0.28	-0.7	-1.21	-0.19	**0.007**	-1.19	-2.35	-0.03	**0.044**
No income (ref)	219	7.3	0											

Note: the modified MCSDS retains 21 of the original 33 items; Civil and Lawful Impression (13 items) and Integrous Image Boosting (8 items) are factors of the modified MCSDS; b represents the standardized coefficient; n and % are the sub-group sample size and weighted percentages; 95% CI indicates the upper and lower bounds of the 95% Confidence Interval; p-values stated in bold are significant at p < .05; Pre-University/Junior College/Vocational Institute/ITE/Diploma are local derivatives of pre-tertiary education

### The association between modified MCSDS, dietary habits, and physical activity

Results from hierarchical linear regression analyses, summarized in Table 4, revealed that higher social desirability bias was significantly associated with healthier self-reported dietary habits (DASH scores), *b* = 0.16, *t* = 4.69, *p* < .001, 95% CI [0.09,0.23], greater physical activity (GPAQ global MET scores), *b* = 73.27, *t* = 1.98, *p*=. 0.048, 95% CI [0.81, 145.73], and shorter sedentary time (GPAQ sedentary score), *b*= -5.48, *t*= -3.76, *p* < .001, 95% CI [-8.34, -2.62], after accounting for sociodemographic and BMI covariates. Additionally, the modified MCSDS explained significant but small proportions of variance in dietary scores (*F*(1, 2556) = 22.00, *R*^*2*^ = 0.1502, step 2 Δ*R*^2^ = 0.015, *p* < .001), physical activity scores (*F*(1, 2556) = 3.93, *R*^*2*^ = 0.0882, step 2 Δ*R*^2^ = 0.0024, *p* = .0475), and sedentary scores, (*F*(1, 2556) = 14.13, *R*^*2*^ = 0.1413, step 2 Δ*R*^2^ = 0.0089, *p* = .0002).

**Table 4:** Summary of hierarchical regression analysis for variables predicting self-reported diet scores (DASH scores), self-reported physical activity scores (GPAQ grand MET score), self-reported sedentary level scores (GPAQ sedentary score)

Variable	b	p value	95% CI	F	R^2^	R^2^ change
*DASH score (step 2)*				22.00	**0.150**	**0.0150**
21-item MCSDS	0.16	**< 0.001**	0.09, 0.23			
*GPAQ grand MET score (step 2)*				3.93	**0.089**	**0.0024**
21-item MCSDS	73.27	**0.048**	0.81, 145.73			
*GPAQ sedentary score (step 2)*				14.1	**0.141**	**0.0089**
21-item MCSDS	-5.48	**< 0.001**	-8.34, -2.62			

note: step 1 comprised of confounding variables, such as age, gender, ethnicity, highest education status, marital status, personal monthly income, and BMI; variable parameters described here belong to the final step of the model; boldface R^2^ change represents significance at *p* < .05; DASH scores refers to the total tally of dietary quality scores in seven categories; GPAQ grand MET score refers to the level of physical activity; GPAQ sedentary score refers to the level of sedentary activity

## Discussion

The main purpose of the study is to ascertain the association between social desirability, sociodemographic, and self-reported lifestyle factors. Prior to the main investigation, the factor structures of the original 33-item MCSDS scale [19] and its popular abbreviated versions [25, 28] were evaluated in the context of a large, representative, multi-ethnic non-western population. A total of nine CFAs were conducted, and the results showed that the full and short-form versions did not achieve adequate fit of data. EFA was subsequently conducted, and a 21-item two-factor solution was ultimately derived. Additional iterations of CFA on the full and split half samples revealed an excellent fit of data for the modified 21-item version. As the dimensions of social desirability are uniquely different in Singapore as compared to elsewhere, it is therefore necessary to discuss its factorial properties in this section.

Overall, the results supported a two-factor structure [26, 30–32]. Like past studies, various items on the full scale had weak rotated factor coefficients (< 0.04), implying that not all items of the original scale had adequately captured the factors [24]. Unique to our investigation, item 1 of the scale, “Before voting in an election, I thoroughly investigate the qualifications of all the candidates”, was removed due to significant concerns raised by participants during the survey, but not due to weak factor loadings. While future research that use this scale may include the first item for scale completeness, users need to be aware that participants may feel uncomfortable answering this question face-to-face and may have to contend with missing values.

The present results differed from Millham’s (1974) components of “attribution” (i.e., tendency to endorse characteristics that are socially desirable; items 1,4,13,17,25,26,27,29) and “denial” (i.e., tendency to deny characteristics that are socially undesirable; items 3,5,9,10,11,23,28)[38, 39], or the two-factor solution of “achievement” and “interpersonal relationship” derived by Verardi and colleagues [27]. Instead, the two factors—Civil and Lawful Impression (CLI) and Integrous Image Boosting (IIB)—appeared to strike a chord with Paulhus’s two components of the Balanced Inventory of Desirable Responding (BDIR; [25]—the concepts of Impression Management (IM), defined as the “conscious dissimulation of test responses designed to create a favourable impression”, and Self-deceptive Enhancement (SDE), defined as “any positively biased response that the respondent actually does believe to be true”. Items that load on the CLI factor corresponded to a mix of IM and SDE items, whereas the items that load on the IIB factor comprised a majority of SDE items with a small overlap of IM items. The highly overlapping constructs of IIB and SDE factors could imply that they were measuring a highly similar construct — an esteem boosting of self-image, whereas the lower degree of overlap between the CLI and the IM factor (or a mixed overlap with both IM and SDE factors) could signify a culturally nuanced form of social self-control that is unique to the present population; one that represents a form of social self-control against the system or an authority. Future research could replicate and extend our findings further by investigating the stability and generalizability of the current factor structure in more stigmatizing fields, such as mental illness perceptions in Singapore [40, 41].

It was discovered that social desirability explained a significant but small proportion of variances of lifestyle factors (physical activity, sedentary time, diet scores), which corroborated past reports that were based in western communities or unrelated to health-based research [12, 42]. The small effect of the modified MCSDS on lifestyle constructs could be due to the study’s emphasis on anonymity and protection of personal data during consent taking, despite it being conducted face-to-face, which could have brought about confidentiality confidence in participants. Older age groups, those of Indian ethnicity, those who were ever married and those without income (as compared to those earning below 2k or at least 10k and above), had a significantly greater tendency to respond in a way that is socially desired. This could be due to unconscious social motivations underlying the need to boost self-image, such as the need for social approval, to avoid negative evaluation, or to be stereotyped against [19], as the surveys were conducted face-to-face. It is thus important for future epidemiology research to take into account these minority and/or vulnerable groups. Improving the level of anonymity, such as by the use of online self-administered questionnaires [43], can further mitigate this bias across groups of individuals who have a greater inclination to give socially desirable answers thus leading to under-estimation of the true extent of the problem in these communities. Gender was not a significant predictor of social desirability scores in the present sample which corroborated the majority of past reports, regardless of MCSDS item makeup [19, 27, 30, 32, 44].

Though a small percentage of variance of dietary intake, physical activity, and sedentary behaviour, were attributed to social desirability bias, not at all accounting for the bias is discouraged. On the contrary, such a bias should be measured and accounted for, ideally, in all health-related research that uses the self-report methodology. Though face-to-face interviewing may contribute to social desirability bias to a greater extent than online-based surveys, this mode of data collection is essential to engage particularly hard-to-reach demographics, such as older respondents who may not be as internet-savvy; therefore, having a mix of online and offline modes of data collection should be encouraged in epidemiological research that requires population representation [45, 46].

### Strengths and limitations

Several caveats have to be taken into consideration before conclusions can be made. This study uses cross-sectional data, therefore, causal or temporal inferences cannot be made. In addition, we did not use objective measures to determine the level of deviation from self -reported physical activity, dietary intake, or sedentary behaviours, and thus, we could not establish the levels of discrepancies in self-reports attributed by the bias. While the response rate of approximately 66% is acceptable, [47], the results may not be representative of non-responders. As this study uses face-to-face surveys, social desirability bias may be larger than other surveying modes that provide better levels of anonymity. However, due to an ageing society, face-to-face interviews were necessary to engage hard-to-reach groups such as older adults. This was the first study that investigated social desirability bias in a multi-cultural Asian context. Results are based on a large cohort sample that is representative of Singapore’s resident population.

## Conclusion

Due to Singapore’s unique cultural makeup, pre-existing popular abbreviated scales had failed to provide an acceptable factorial fit of local data. Instead, a unique 21-item modified scale had produced the best fit. Additionally, certain groups of participants were identified to be more likely to exercise this bias, which could be due to unconscious social motivations that interact with face-to-face research. Nonetheless, only a small proportion of variance of scores on self-report lifestyle scores could be explained by social desirability bias, which is reassuring as it suggests that participants were not greatly influenced by social expectations to respond in a socially desirable way despite a face-to-face survey, which has a lower level of anonymity than online surveys. In conclusion, measuring social desirability bias in health-based research reliant on self-report methodology is highly encouraged so as to account for its effects.

## Data Availability

The data that support the findings of this study are available but restrictions apply to the availability of these data, which were used under license for the current study, and so are not publicly available. Data are however available from the authors upon reasonable request and with permission; data requests can be submitted to mythily@imh.com.sg.

## List of Abbreviations

KAP: Knowledge, Attitudes, and Practices
IRRC: Institutional Research Review Committee
DSRB: Domain Specific Review Board
MCSDS: Marlowe–Crowne Social Desirability Scale
GPAQ: Global Physical Activity Questionnaire
WHO: World Health Organisation
METs: Metabolic Equivalents
GPAQ grand MET score: Global MET score
GPAQ sedentary score: Sedentary MET score
DASH: Dietary Approaches to Stop Hypertension
RMSEA: Root mean square error of approximation
CFI: Comparative fit index
TLI: Tucker–Lewis index
EFA: Exploratory factor analysis
CFA: Confirmatory factor analysis
ULSMV: Unweighted least square mean and variance
